# Humidity-Sensitive PMMA Fiber Bragg Grating Sensor Probe for Soil Temperature and Moisture Measurement Based on Its Intrinsic Water Affinity

**DOI:** 10.3390/s21216946

**Published:** 2021-10-20

**Authors:** Heng Wang, Shixin Gao, Xiaoyu Yue, Xin Cheng, Qi Liu, Rui Min, Hang Qu, Xuehao Hu

**Affiliations:** 1 College of Science, Shenyang Aerospace University, Shenyang 110136, China; wangheng@sau.edu.cn (H.W.); gaoshixin@stu.sau.edu.cn (S.G.); 2 Research Center for Advanced Optics and Photoelectronics, Department of Physics, College of Science, Shantou University, Shantou 515063, Guangdong, China; 19xyyue@stu.edu.cn (X.Y.); haqux@stu.edu.cn (H.Q.); 3 Key Laboratory of Intelligent Manufacturing Technology of MOE, Shantou University, Shantou 515063, Guangdong, China; 4 Photonics Research Centre, Department of Electrical Engineering, The Hong Kong Polytechnic University, Kowloon, Hong Kong 999077, China; eechengx@polyu.edu.hk; 5 National Engineering Laboratory for Crop Efficient Water Use and Disaster Mitigation, Key Laboratory of Prevention and Control of Residual Pollution in Agricultural Film, Ministry of Agriculture and Rural Affairs, Institute of Environment and Sustainable Development in Agriculture, Chinese Academy of Agricultural Sciences, No. 12 Zhongguancun South Street, Beijing 100081, China; liuqi@caas.cn; 6 Center for Cognition and Neuroergonomics, State Key Laboratory of Cognitive Neuroscience and Learning, Beijing Normal University at Zhuhai, Zhuhai 519087, Guangdong, China; ruimin@bnu.edu.cn

**Keywords:** polymer optical fiber, fiber Bragg grating, PMMA, soil moisture content, temperature, humidity

## Abstract

Soil moisture measurement is very important for soil system monitoring. Compared to the traditional thermo-gravimetric technique, which is time-consuming and can be only performed in labs, the optic-fiber technique has unique advantages, such as small size, remote application in fields, fast response time and immunity to electromagnetic fields. In this paper, the soil moisture is measured by using a polymer optical fiber Bragg grating (POFBG) probe with a packaged dimension of 40 mm × 15 mm × 8 mm. Due to the intrinsic water-absorbing property of poly (methyl methacrylate) (PMMA), optical fiber Bragg gratings based on PMMA have been widely investigated for humidity measurement. Taking advantage of this, a sensor based on the POFBG is investigated to verify the soil condition. The POFBG is protectively integrated inside a stainless-steel package. A window is opened with a thin polypropylene mat as a filter, which allows the air to go through but prevents the soil from going inside to pollute the POFBG. The sensor probe is embedded in soils with different gravimetric soil moisture contents (SMCs) ranging from 0% to 40% and, then, insulated by polyethylene films to minimize the impact from the external environment, showing an average temperature cross sensitivity of −0.080 nm/°C. For a constant temperature, an exponential relationship between the Bragg wavelength and the SMC is obtained. For the SMCs between 8% and 24%, linear relationships are presented showing a temperature-corresponded sensitivity between 0.011 nm/% and 0.018 nm/%. The maximal sensitivity is calculated to be 0.018 nm/% at 20 °C, which is 28 times as high as that in the previous work. For the SMC over 24%, the sensor becomes insensitive because of humidity saturation in the cavity of the sensor probe. Though temperature cross sensitivity is problematic for SMC measurement, the influence could be eliminated by integrating another humidity-insensitive temperature sensor, such as a silica FBG temperature sensor.

## 1. Introduction

Soil moisture plays an important role in the soil system, which mainly contains soil minerals, moisture and air [1]. Measurements of soil moisture content (SMC) are always required to study the biological, hydrological and geological properties of soils. Growth of plants, organization of the natural ecosystems and biodiversity are also related to SMC. Furthermore, the evolution of the SMC corresponds to topography, landscape position, slope, vegetation, soil structure and texture and architectonic structure above the soil. Especially in agriculture, timely regulation of SMC is vital for plant irrigation [2].

Various methodologies in labs or in situ have been adopted for the SMC measurements. The classical method to measure SMC is the so-called thermo-gravimetric technique, which serves as a standard reference for the determination of the SMC [3]. In this technique, the soil moisture is removed from the soil sample by evaporation. Normally, this measurement is performed using an oven that dries a wet-soil sample with a weight of 100 g or less. After drying for 24 h at 105 °C without further significant weight loss, the weight of the dry soil sample is first recorded [4]. The amount of moisture in percentage in the soil is usually defined as gravimetric SMC, w, which represents the ratio of the mass of moisture in the soil sample to the mass of the dried soil, or defined as volumetric SMC, v, which represents the ratio of the volume of moisture present in the soil to the volume of the dried soil. The volumetric SMC is related to the gravimetric SMC by:

(1) $$v=w\left(\rho_{s}/\rho_{w}\right)$$

where $\rho_{s}$ is the density of the dried soil, and $\rho_{w}$ is the density of the moisture [3]. However, this method for SMC measurement is time consuming and can be only performed in labs.

Different from the classical technology, modern soil moisture measurement techniques, such as neutron scattering [5], dielectric [6], electrical impedance [7], micro electro mechanical system [8] and thermal dissipation [9], can be employed not only in labs but also in situ to obtain the amount of moisture in several minutes to several hours [2]; however, the sensor probes based on these methods operate with electric power, and thus, the electromagnetic interference exists. Besides, it is impossible to realize distributed or quasi-distributed remote measurements in the field without the deployment of the Internet of Things (IoT), which may be interfered by electromagnetic waves as well.

An alternative technique for SMC measurements is based on the optical route that characterize variations in optical intensities or spectra, as the light goes through the soil. The optical technique mainly includes two types, i.e., the polarized light technique and the fiber-optic technique. The operation principle for the former is that existence of moisture on the surface of the soil can cause polarization in the reflected beam; however, operation of this method is influenced by the soil type and the roughness of the soil surface [10]. Optic-fiber sensors have been widely used for the detection of physical and chemical parameters because of the advantages, such as the immunity to electromagnetic fields, small size and remote applications [11]. Here, the principle for the fiber-optic technique is normally based on modified optical fibers. Light attenuation in an unclad fiber varies with the critical angle of internal reflection, which corresponds to the surrounding refractive index as a function of the SMC [12]. However, this technique is difficult to use in distributed sensing applications. Recently, a silica fiber Bragg grating (FBG)-based sensor including resistance wires for heating was characterized for indirect volumetric SMC measurement showing an average measurement accuracy of 1.5%. However, this is not a pure optical fiber sensor, and it can be interfered by electromagnetic fields [13]. Leone et al. proposed another SMC sensor with two silica FBGs embedded in a tube, one of which was coated with polyimide for water adsorption. The volumetric SMC corresponds to the relative humidity (RH) in the tube. However, this sensor probe is very bulky with a package volume of 800 cm^3^, which is not convenient to operate [14].

Compared to silica optical fibers, poly (methyl methacrylate) (PMMA) polymer optical fibers (POFs) have unique advantages, such as higher failure strain (up to 100%), lower Young’s modulus (~3.3 GPa), an affinity for water (up to 2%) [15], larger absolute value of thermo-optic coefficient (−8.5 × 10^−5^) [16] and better biocompatibility [17]. The first FBG in the PMMA POF was fabricated in 1999 [18], and since then, polymer optical fiber Bragg gratings (POFBGs) have been manufactured in different materials, such as polycarbonate [19], Zeonex [20], Topas [21] and CYTOP [22]. Based on the advantages of POFs in different materials, POFBGs have been used for various applications, such as sensing of temperature [23], humidity [19,23,24], pressure [20,25], strain [26], accelerometry [27] and physiological fluids [28]. Nonetheless, PMMA is the most commonly used material for POFBG fabrication [29,30,31] due to ease of handling and processing and low cost. The PMMA FBG could be fabricated with a single pulse at 266 nm using phase mask technique and could achieve a stable grating reflectivity of ~78% after post-annealing [32]. Though polycarbonate (PC) is able to absorb water for humidity sensing, the saturation value is only 0.3% at 23 °C [19], much smaller than that for PMMA. PMMA is the most popular material for humidity-based sensing application due to its water affinity property [33].

Though PMMA-based POFBGs have been widely characterized for both humidity and temperature measurement [23], it is more important for them to be investigated in practical applications based on their unique properties, such as large water affinity [15] and large thermal-optic coefficient [16]. Thanks to PMMA water affinity, Zhang et al. demonstrated a saline solution concentration sensor based on a POFBG. The absorbed water content in the fiber depends on the concentration because of the osmotic effect [28]. In this work, based on the inherent high water affinity of PMMA material, we experimentally demonstrate a sensor probe with POFBG encapsulated inside the stainless-steel package. Both SMC and temperature characterization are carried out, and the sensor shows a sensitivity of −0.080 ± 0.003 nm/°C by linear regression in different soils and an exponential relationship between Bragg wavelength and SMC. The proposed sensor probe, featuring compact size, good reversibility and stability, is appealing for distributed and remote soil condition sensing applications.

## 2. Principle and Experimental Set-Up

### 2.1. Principle of Soil Moisture Sensing

The soil moisture investigation is based on the FBG technique [34]. A uniform FBG is a periodic and permanent modulation of the refractive index in the fiber core along the fiber axis. An FBG works as a mirror reflecting light around the so-called Bragg wavelength $\lambda_{B}$ that is a function of the effective refractive index of the fiber core mode ($n_{\mathrm{eff}}$) and the modulation period (Λ). The first order Bragg grating wavelength is given by

(2) $$\lambda_{B}=2n_{\mathrm{eff}}\Lambda$$

For PMMA POFBGs, it is well known that the thermo-optic coefficient of PMMA is negative, and it contributes to the Bragg wavelength shift more than the thermal expansion coefficient [35]. The water affinity of PMMA induces not only a swelling of the POF but also an increase in refractive index [36,37], both of which contribute to an increase in the Bragg wavelength of a POFBG inscribed in the fiber. Thus, for a POFBG, both $n_{\mathrm{eff}}$ and Λ can be influenced by soil temperature T and w (gravimetric SMC). The latter corresponds to the humidity around the fiber. By making first order derivatives of Equation (2), Bragg wavelength $\lambda_{B}$ can be expressed as

(3) $$\frac{\partial \lambda_{B}}{\partial T}=2n_{\mathrm{eff}}\frac{\partial \Lambda }{\partial T}+2\Lambda \frac{\partial n_{\mathrm{eff}}}{\partial T}=\lambda_{B}\left(\alpha_{\Lambda }+\alpha_{n_{\mathrm{eff}}}\right)$$

(4) $$\frac{\partial \lambda_{B}}{\partial w}=2n_{\mathrm{eff}}\frac{\partial \Lambda }{\partial w}+2\Lambda \frac{\partial n_{\mathrm{eff}}}{\partial w}=\lambda_{B}\left(\beta_{\Lambda }+\beta_{n_{\mathrm{eff}}}\right)$$

where

(5) $$\alpha_{\Lambda }=\left(1/\Lambda \right)\left(\partial \Lambda \right)/\left(\partial T\right)$$

(6) $$\alpha_{n_{\mathrm{eff}}}=\left(1/n_{\mathrm{eff}}\right)\left(\partial n_{\mathrm{eff}}\right)/\left(\partial T\right)$$

(7) $$\beta_{\Lambda }=\left(1/\Lambda \right)\left(\partial \Lambda \right)/\left(\partial w\right)$$

(8) $$\beta_{n_{\mathrm{eff}}}=\left(1/n_{\mathrm{eff}}\right)\left(\partial n_{\mathrm{eff}}\right)/\left(\partial w\right)$$

Here, $\alpha_{\Lambda }$ represents the coefficient of thermal expansion, $\alpha_{n_{\mathrm{eff}}}$ represents the normalized thermal-optic coefficient, $\beta_{\Lambda }$ represents the coefficient of hygral expansion induced by SMC change, and $\beta_{n_{\mathrm{eff}}}$ represents the normalized coefficient of refractive index variation induced by SMC shift. In order to simplify the equations and reduce the sensor response time, a pre-strain can be applied to POFBGs [35,38], and consequently, the coefficients of thermal and hygral expansion are ignored. Finally, we assume that the Bragg wavelength is related to soil T and w, as well as $\alpha_{n_{\mathrm{eff}}}$ and $\beta_{n_{\mathrm{eff}}}$.

### 2.2. POFBG Fabrication and Experimental Set-Up

The PMMA POFs used in this work were fabricated by “pull-and-through” technique at *The Hong Kong Polytechnic University*. The diameters of the core and cladding are 5.6 μm and 125 μm, respectively [25,35]. The core consists of PMMA doped with benzyl dimethyl ketal of 1 wt.% in concentration, which is not only used to increase the refractive index for lighting guiding but also elevate the photosensitivity for FBG inscriptions. Before grating inscription, the POF was annealed at 80 °C for 48 h to release the frozen-in stress for a better inscription stability [39] and connected to silica optical fiber pigtails by UV glue [40]. Then, the 4 mm-long POFBG was successfully inscribed by a UV pulsed laser using phase mask technique similar to that reported in our previous work [32]. The UV pulsed laser with an emitting wavelength of 266 nm has a pulse width of ~6 ns and a pulse energy of 1.4 mJ. The phase mask used in this work is optimized at the same wavelength with a uniform pitch of 1060 nm. A cylindrical lens with a focal length of 3 cm was used to focus the beam along the fiber core to increase the power density. To alleviate the heat effect and avoid heat accumulation, the laser irradiation frequency is reduced to 1/6 Hz [41]. The POFBG was obtained after 15 pulses of laser irradiation. Afterwards, the grating was post-annealed at 80 °C for 24 h to increase the POFBG stability [32]. The grating spectrum was measured by the FS22SI Industrial BraggMETER Interrogator (HBM FiberSensing S.A., Moreira, Portugal) with a resolution of 1 pm and a scanning rate of 1 Hz.

**Figure 1:**
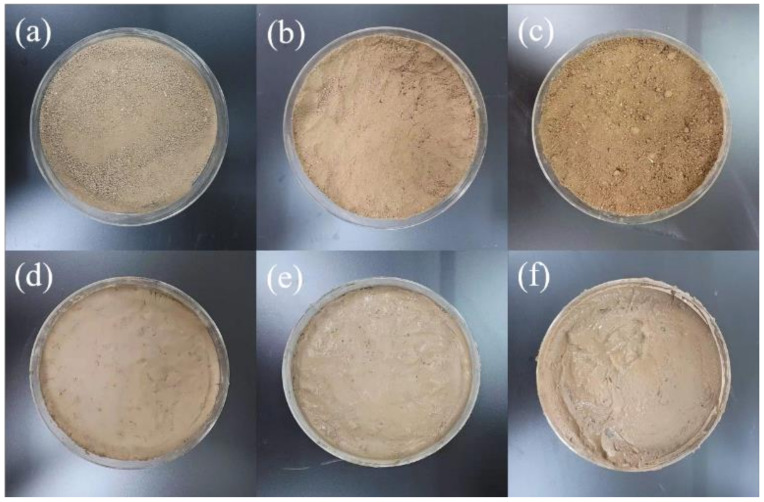
Images of soils with different SMCs (0% (**a**), 8% (**b**), 16% (**c**), 24% (**d**), 32% (**e**) and 40% (**f**)), prepared by mixing the dried soil (in an oven at a temperature of 105 °C for 24 h) with deionized water in different weights.

The soils with different moisture contents were prepared by two steps. First, the soil was dried in an oven at a temperature of 105 °C for 24 h. Then, 100 g (or 92 mL) of the dried soil was mixed with deionized water in different weights, i.e., 0 g, 8 g, 16 g, 24 g, 32 g and 40 g, respectively, in a petri dish. Thus, five soil samples were prepared with different gravimetric SMCs, which were 0%, 8%, 16%, 24%, 32% and 40%, respectively, as depicted in Figure 1.

**Figure 2:**
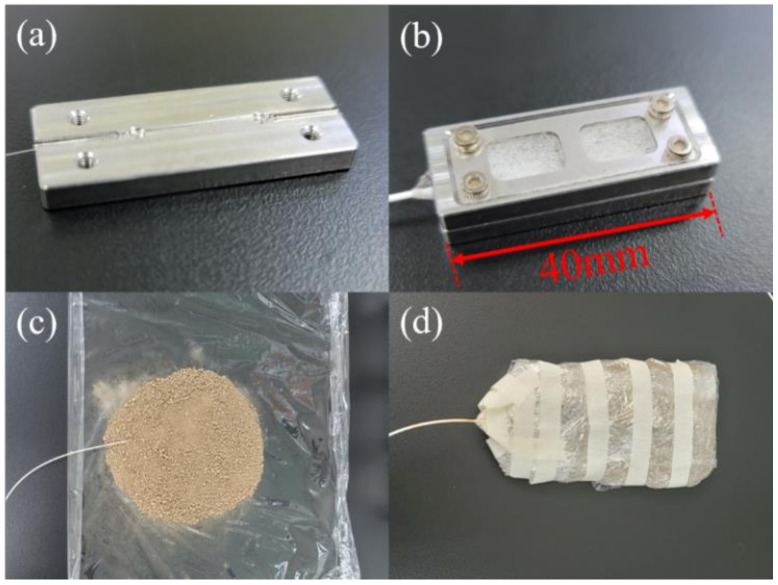
Installation of the experimental set-up. (**a**) The POFBG positioned in the V-groove; (**b**) sensor probe: the POFBG in the stainless-steel package with polypropylene mat as a filter; (**c**) the sensor probe embedded in soils; (**d**) sensor probe wrapped up by polyethylene films.

**Figure 3:**
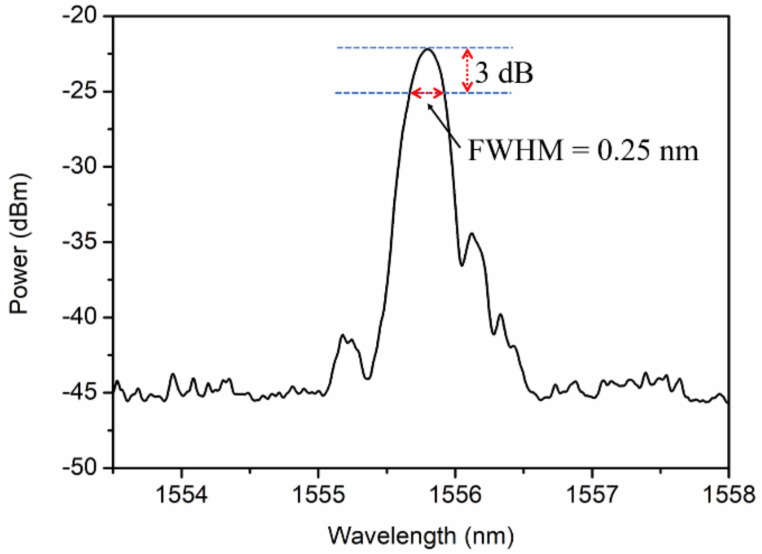
Reflected spectrum of the POFBG after annealing at 60 °C and 90% RH for 48 h. The full width at half maximum is 0.25 nm.

Afterwards, a pre-strain of 0.22% was applied before the UV curing of the POFBG in the V-groove of a customized protection package, as shown in Figure 2a. The package (sensor probe) with a window was made of stainless-steel with a dimension of 40 mm × 15 mm × 8 mm with a small cavity. The window was covered by a thin polypropylene mat, which acted as a filter to allow the air to go through but prevented the soil from going inside to pollute the POFBG, as depicted in Figure 2b. Then, the POFBG was annealed at 60 °C and 90% RH for 48 h to further improve the performance of the POFBG in terms of stability especially in humidity measurement [24]. The reflected spectrum was shown in Figure 3 with a full width at half maximum (FWHM) of 0.25 nm. After that, the sensor probe was embedded in soils with different gravimetric soil moisture contents (SMCs) from 0% to 40%, as demonstrated in Figure 2c. After that, the sensor probe was wrapped up by polyethylene films to be insulated without the influence of outside humidity, as displayed in Figure 2d. Because of the high transmission losses of PMMA POFs at ~1550 nm, one could inscribe FBGs at ~850 nm by a phase mask with a proper pitch [42], so that the transmission losses could be reduced [43,44].

## 3. Experimental Results and Discussions

### 3.1. Temperature Measurement in Soils with Different SMCs

**Figure 4:**
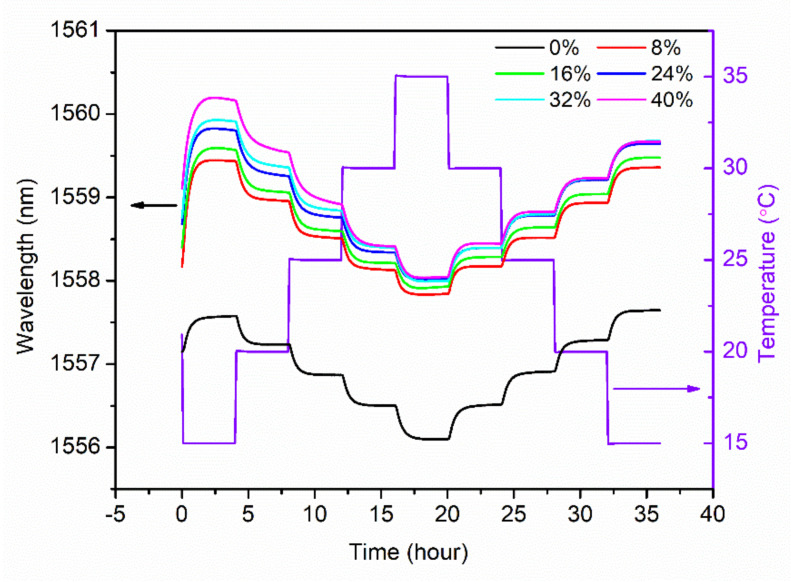
Evolutions of Bragg wavelength in terms of temperature between 15 °C and 35 °C with a step of 5 °C (increase and decrease). This experiment was conducted for a series of soils with different SMCs (0%, 8%, 16%, 24%, 32% and 40%).

**Figure 5:**
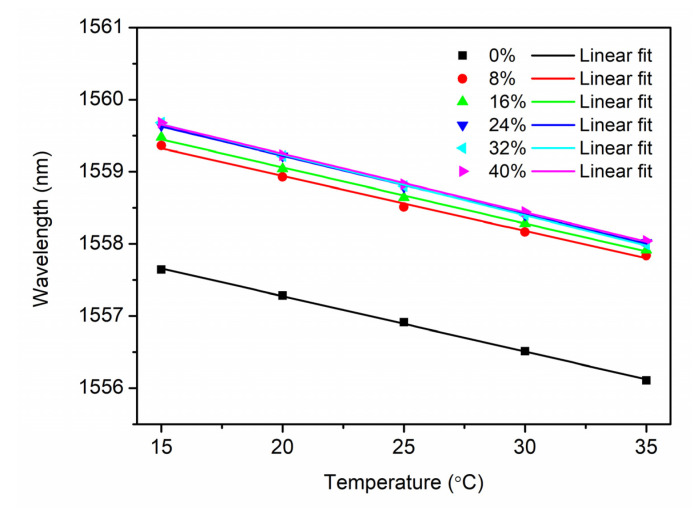
Linear fit of Bragg wavelength versus temperature in the temperature decrease process for soils with different SMCs (0%, 8%, 16%, 24%, 32% and 40%) with sensitivities (−0.077 nm/°C, −0.076 nm/°C, −0.078 nm/°C, −0.081 nm/°C, −0.084 nm/°C and −0.081 nm/°C), respectively.

For different SMCs from 0% to 40%, temperature characterization of the sensor probe was implemented for one cycle between 15 °C and 35 °C with a step of 5 °C and a duration of 4 h. Figure 4 shows the evolution of the Bragg wavelength with respect to the temperature changes in 36 h. For the SMC at 0%, the Bragg wavelength during the whole process is much lower than those for SMCs from 8% to 40%, which may be caused by very high extraction of water in the extremely dry conditions. It is also found that as the temperature increases, the Bragg wavelengths are not completely stabilized within 4 h, especially for SMC 24%, 32% and 40%, which may be attributed to the longer moisture equilibrium time among the soil, the air in the cavity of the package and the POF. However, as the temperature decreases, it appears that the Bragg wavelengths are stabilized sooner, as shown in Figure 4. Thus, compared to the increase process, the temperature measurement in the decrease process is more reliable. The corresponding response time defined as 90% of the total Bragg wavelength shift is achieved, which is ~1 h. After linear regression in the temperature decrease process, the relationship between the Bragg wavelength and the temperature is illustrated in Figure 5, showing sensitivities of −0.077 nm/°C, −0.076 nm/°C, −0.078 nm/°C, −0.081 nm/°C, −0.084 nm/°C and −0.081 nm/°C for SMC 0%, 8%, 16%, 24%, 32% and 40%, respectively. These sensitivities are very similar, and the average value is −0.080 ± 0.003 nm/°C.

**Figure 6:**
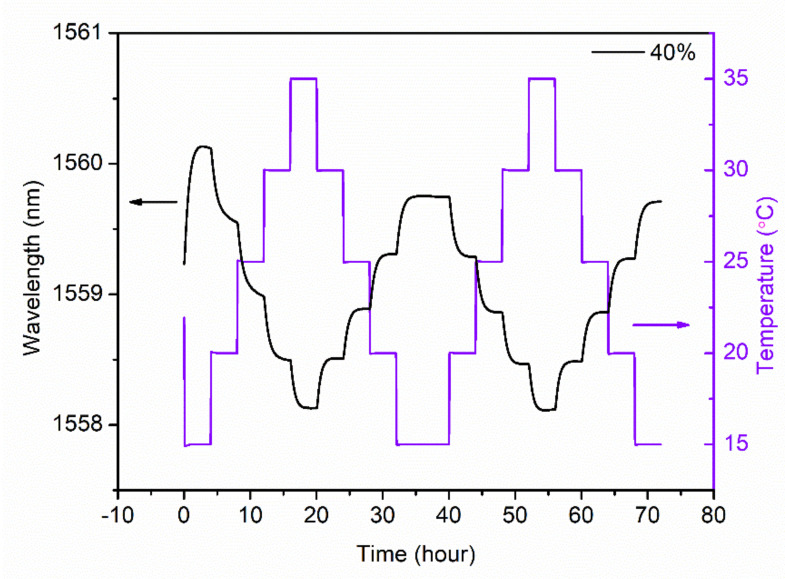
Bragg wavelength shift as a function of temperature in the range 15 °C–35 °C in two cycles for SMC 40%.

To further verify the reverse performance of the sensor probe in terms of temperature measurement, two cycles of temperature characterization were conducted for SMC 40%, as shown in Figure 6. It is found that the Bragg wavelength evolutions in the temperature decrease process of cycle 1 and in the temperature increase process of cycle 2 are reversible, and the Bragg wavelength evolutions in the temperature decrease processes of cycle 1 and cycle 2 are highly coincident. These two phenomena further confirm that the data obtained in the temperature decrease process are reliable for temperature characterization.

### 3.2. Bragg Wavelength Versus SMC

**Figure 7:**
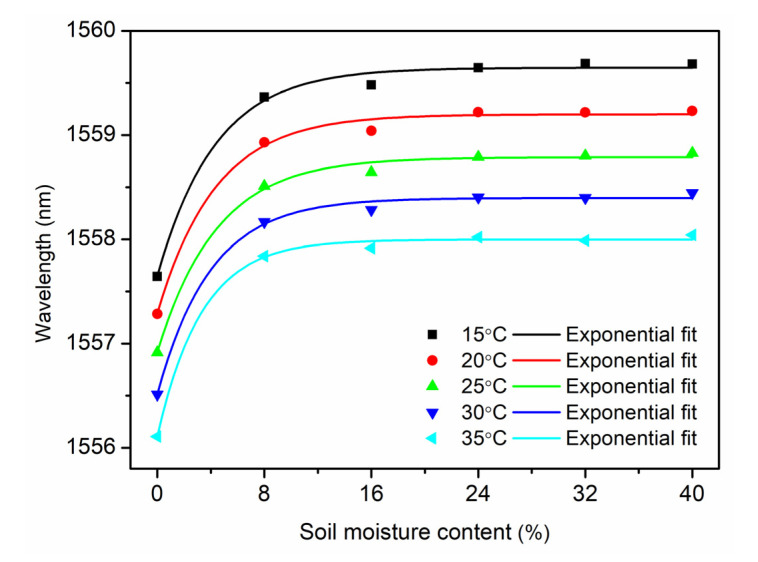
Bragg wavelength versus SMC at a constant temperature from 15 °C to 35 °C with a step of 5 °C.

**Figure 8:**
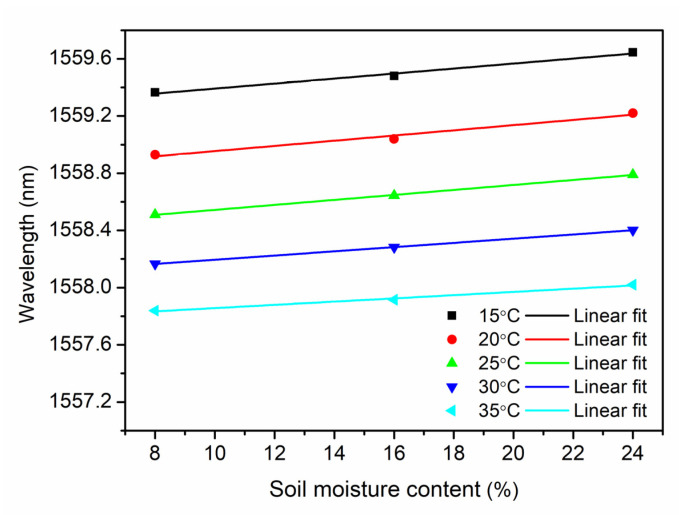
Linear fit of Bragg wavelength versus SMC from 8% to 24% at a constant temperature from 15 °C to 35 °C with a step of 5 °C.

Based on the data from the temperature decrease process of the first cycle (see Figure 4), the relationship between the Bragg wavelength and the SMC at a constant temperature, which is 15 °C, 20 °C, 25 °C, 30 °C and 35 °C, respectively, is demonstrated in Figure 7. Using exponential fitting, a non-linear relationship is displayed. It is found that the sensitivity is decreasing at higher SMCs, and it becomes even insensitive for the SMC over 24%, which is similar to the report by Leone et al. [14]. Though the greatest variation was obtained in the range from 0 to 8% showing highest SMC sensitivities, this range is far from the threshold definition of the dry soil in agriculture, which is 10~15% depending on the soil composition. However, the sensor operating range from 8% to 24% is helpful for a warning of a dry soil, which needs to be further investigated. Additionally, the sensor seems to be practically insensitive for a SMC range between 16% and 24% by the exponential fitting, which is worse than the result obtained in [14]. Fortunately, for gravimetric SMC between 8% and 24% (or 8.7% and 26.1% in volumetric SMC), a linear relationship is obtained by a linear regression of raw data from Figure 7 with sensitivities between 0.011 nm/% and 0.018 nm/%, as shown in Figure 8.

**Figure 9:**
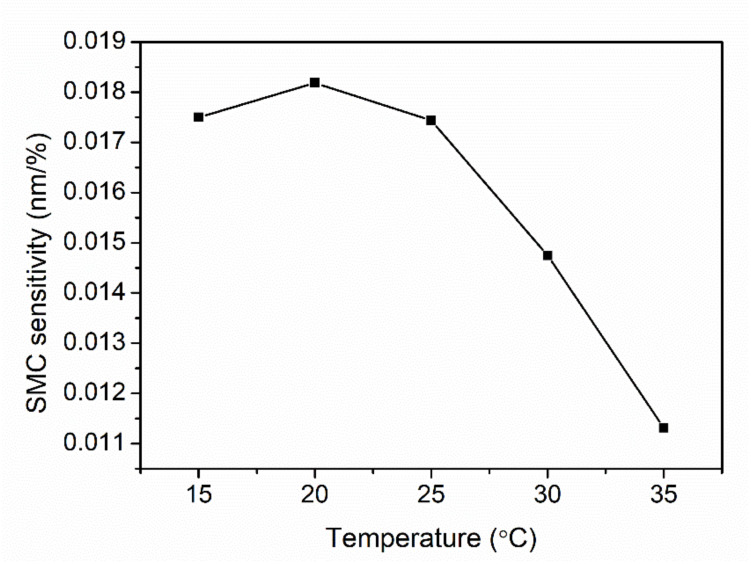
SMC sensitivities calculated in the SMC range between 8% and 24% as a function of temperature change from 15 °C to 35 °C with a step of 5 °C.

The sensitivities at different temperatures are displayed in Figure 9. The maximal sensitivity appears at 20 °C, and then, the sensitivity decreases down to 0.011% when the temperature increases. This phenomenon agrees with the investigation in deuterated PMMA material, whose refractive index humidity dependence decreases as temperature increases in the range 20 °C to 40 °C [36]. The maximal sensitivity 0.017 nm/% in volumetric SMC is 28 times as high as 0.0006 nm/% in the range 14–37% in volumetric SMC carried out by Leone et al. [14]. For the SMC over 24%, the insensitivity of the sensor probe could be attributed to the humidity saturation in the cavity of the sensor probe. However, the average real SMC is around 20% up to 35%. To break the limit due to the humidity saturation at higher SMCs in this work, a top-sealed tube with a micro-porous membrane at the bottom could be vertically buried in the soil. Water molecules in the vapor state in the soil could pass through the membrane and disperse inside the tube. It has been reported that because of RH stratification effect, RH decreases with the height inside the tube. Based on this, our sensor probe could be positioned at a higher level inside the tube to avoid the moisture saturation at higher SMCs. Finally, the sensing range of the SMC could be extended.

The system, as developed in this work, is supposed to go to the field, be buried and keep measuring SMC, which, however, varies during raining and dry seasons, making the SMC increase and decrease. Thus, it is important to evaluate the sensor response time with respect to weather change. Thanks to the pre-strain of the fiber, the swelling-induced wavelength change is eliminated [38]. Consequently, the wavelength change in the grating is resulted from the refractive index change in the fiber core, and the response time due to the humidity changes (related to the SMC) depends on the diffusion coefficient of water into the PMMA core and the geometric structure of the POF [37]. According to the results reported in the references [37,38], the response time with ±10%RH change is estimated to be less than 20 min.

It is worth mentioning that as shown in Figure 6 when the temperature varies from 15 °C to 35 °C, the wavelength varies about 2 nm, and thus, the variation would completely mask the wavelength change due to the SMC change in condition that the sensor probe is applied in the field without temperature control. Though temperature cross sensitivity is problematic for the SMC measurement, the influence could be eliminated by integrating another humidity-insensitive temperature sensor, such as a silica FBG temperature sensor, as a reference.

## 4. Conclusions

In summary, based on the inherent water-absorbing property of PMMA, a POFBG sensor probe was investigated for SMC measurements in the range from 0% to 40% with an interval of 8%. For each SMC, temperature characterization was conducted from 15 °C to 35 °C with a mean sensitivity of −0.080 nm/°C. For a constant temperature, an exponential relationship was turned out between the Bragg wavelength and the SMC with decreasing sensitivity. For the SMCs between 8% and 24%, linear relationships were obtained between 0.011 nm/% and 0.018 nm/%. The maximal sensitivity 0.018 nm/% appears at 20 °C, which is 28 times as high as that in the previous work. For the SMC over 24%, the sensitivity declines to almost zero due to the humidity saturation in the cavity. The proposed optical fiber sensor has advantages of capability of distributed remote application, compact size, good reversibility and stability, which make it attractive for soil-condition monitoring in real time. In the near future, both SMC measurement and temperature characterization will be performed in the field. Afterwards, an extra humidity-insensitive temperature sensor based on a silica FBG could be integrated into this POFBG-based sensing system, so that both temperature and SMC are able to be presented in this system at the same time.
